# Epidural stimulation restores muscle synergies by modulating neural drives in participants with sensorimotor complete spinal cord injuries

**DOI:** 10.1186/s12984-023-01164-1

**Published:** 2023-05-03

**Authors:** Rajat Emanuel Singh, Aliya Ahmadi, Ann M. Parr, Uzma Samadani, Andrei V. Krassioukov, Theoden I. Netoff, David P. Darrow

**Affiliations:** 1 Department of Biomedical Engineering, University of Minnesota, Minneapolis, MN USA; 2 Department of Kinesiology, Northwestern College, Orange, IA USA; 3 Division of Neurosurgery, Hennepin County Medical Center, Minneapolis, MN USA; 4 Department of Neurosurgery, University of Minnesota, Minneapolis, MN USA; 5 Department of Bioinformatics & Computational Biology, UMN, Minneapolis, MN USA; 6 Minneapolis Veteran Affairs Medical Center, Minneapolis, MN USA; 7 International Collaboration on Repair Discoveries (ICORD), University of British Columbia (UBC), Vancouver, Canada; 8 Division of Physical Medicine & Rehabilitation, Department of Medicine, UBC, British Columbia , BC Canada; 9 GF Strong Rehabilitation Center, Vancouver Coastal Health, Vancouver, BC Canada

**Keywords:** Muscle synergies, Spinal Cord Injury, Brain motor control assessment, Electromyography, Complexity

## Abstract

Multiple studies have corroborated the restoration of volitional motor control after motor-complete spinal cord injury (SCI) through the use of epidural spinal cord stimulation (eSCS), but rigorous quantitative descriptions of muscle coordination have been lacking. Six participants with chronic, motor and sensory complete SCI underwent a brain motor control assessment (BMCA) consisting of a set of structured motor tasks with and without eSCS. We investigated how muscle activity complexity and muscle synergies changed with and without stimulation. We performed this analysis to better characterize the impact of stimulation on neuromuscular control. We also recorded data from nine healthy participants as controls. Competition exists between the task origin and neural origin hypotheses underlying muscle synergies. The ability to restore motor control with eSCS in participants with motor and sensory complete SCI allows us to test whether changes in muscle synergies reflect a neural basis in the same task. Muscle activity complexity was computed with Higuchi Fractal Dimensional (HFD) analysis, and muscle synergies were estimated using non-negative matrix factorization (NNMF) in six participants with American Spinal Injury Association (ASIA) Impairment Score (AIS) A. We found that the complexity of muscle activity was immediately reduced by eSCS in the SCI participants. We also found that over the follow-up sessions, the muscle synergy structure of the SCI participants became more defined, and the number of synergies decreased over time, indicating improved coordination between muscle groups. Lastly, we found that the muscle synergies were restored with eSCS, supporting the neural hypothesis of muscle synergies. We conclude that eSCS restores muscle movements and muscle synergies that are distinct from those of healthy, able-bodied controls.

**Supplementary Information:**

The online version contains supplementary material available at 10.1186/s12984-023-01164-1.

## Introduction

Around 790,000 people suffer a traumatic spinal cord injury (SCI) every year worldwide [1], and within the U.S.A., approximately 23.6% of the SCI population has motor and sensory complete paralysis [2]. Patients who have not regained motor control after one year rarely go on to do so and are considered to have chronic SCI [3]. Exercise or activity-based therapies (locomotor and non-locomotor training) with functional electrical stimulation, which rely on residual ascending pathways [4–7], remain the most effective treatments in such cases, especially for incomplete SCI [8]. However, motor and sensory complete SCI patients lack functional neural pathways that can be utilized to train and restore volitional control through exercise or activity-based therapies.

Motor and sensory complete SCI patients have an American Spinal Injury Association (ASIA) Impairment Score (AIS) A. The Epidural Stimulation After Neurological Damage (ESTAND) clinical study has shown that epidural spinal cord stimulation (eSCS) in AIS A patients partially restores voluntary motor function [9, 10]. In this study, we found a measurable improvement in volitional control in all six SCI participants with AIS A, and in some cases, the SCI participants demonstrated movement control without active stimulation after long-term eSCS [9]. To measure the recovery of motor control, we employed a standardized surface electromyography(sEMG)-based brain motor control assessment (BMCA) with and without stimulation. Our BMCA protocol incorporated such tasks as relaxation, reinforcement maneuvers (deep breath, neck flexion, Jendrassik maneuver, and bi-lateral shoulder shrug), and voluntary leg movements (bilateral (BL) hip flexion/extension, isolated hip flexion/extension of left and right side, BL ankle dorsiflexion/plantarflexion, isolated dorsiflexion/plantarflexion of the left and right foot.). The changes in motor control due to tonic neuromodulation measured during the BMCA task afforded us an opportunity to measure neuroplasticity in humans. We previously reported that despite ongoing optimization, the total sEMG activity of the legs seemed to plateau and even decrease after the first six months even though a subjective improvement in motor control was achieved [9, 11]. As a result, we endeavored to objectively characterize changes in neuromuscular control associated with spinal cord plasticity over time with eSCS therapy to better elucidate this discrepancy.

We propose to use muscle synergy analysis [12] and complexity analysis [13, 14] to quantify changes in sEMG patterns during participants’ recoveries. Muscle synergies are considered to have a modular organization in the CNS, which, when activated by neural drives, forms a movement. Thus, synergies and the associated neural drives can explain the neurophysiological characteristics of a movement [12]. Furthermore, we map muscle activity to the spinal cord (rostro-caudal plane) to estimate how activation within the spinal cord changes with stimulation over time [15].

There are two competing hypotheses on the origin of muscle synergies, one suggesting a neural basis and the other a task-dependent basis. The task-based synergy hypothesis states that synergies, which are low-dimensional modules arising from regularities in sEMG signals, are determined by the motor tasks or generated by feedback-driven activities. These synergies can result from fixed muscle length changes due to anatomy [16, 17]. For example, a cadaveric model lacking a central controller demonstrated coupling between muscles due to motor tasks, which is considered task-dependent behavior. Hence, in the task-dependent hypothesis, changes in the synergies reflect changes in the dynamics of the task, limb biomechanics, and/or musculoskeletal structure [16, 17].

The neural synergy hypothesis states that changes in muscle synergies (number/dimensionality and structure) reflect neuromodulatory changes directly mediated by the CNS [12, 18]. The dimensionality and structure of synergies are generally preserved for natural motor behavior regardless of task and/or musculoskeletal structure and may only change during new skill acquisition. Therefore, in this hypothesis, limiting or blocking the sensory input does not alter the synergy structure or dimensionality of a natural motor behavior [19], and synergies are considered to be centrally organized and activated through spinal and supraspinal commands. A general experimental approach to demonstrate the neural basis of muscle synergy stimulates the spinal cord and examines the consistency in natural motor behavior patterns [16, 18].

Several studies have tested and validated these hypotheses using participants with and without movement disorders [20]. However, it has been very difficult to disambiguate the two hypothesized origins of muscle synergies in humans because it is challenging to do the same task in participants with and without proper neural control. Therefore, restoring neural control using eSCS in participants with motor and sensory complete SCI provides a unique opportunity to study the same task with and without neural control, which allows us to directly address the origin of muscle synergies. We hypothesize that muscle synergies have a neural origin and believe this is the first study to provide direct evidence for their neural basis in humans. In addition, this framework allows us to examine the effect of long-term epidural stimulation on muscle synergies during the recovery of participants with motor and sensory complete SCI, which we hypothesized would demonstrate improvements but with distinct divergence from the synergies of able-bodied control participants.

## Material and methods

### Participant recruitment/description

**Fig. 1:**
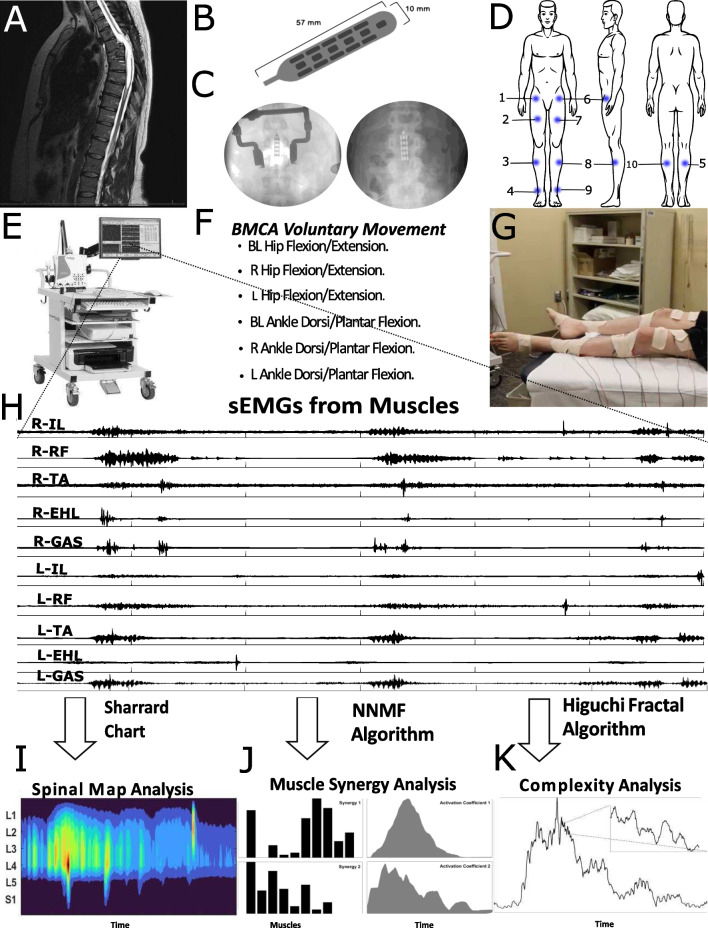
Experimental design and protocol. **A** MRI scan of an SCI participant in this study with a severe injury in the T4/T5 section. **B** Abbott TripoleTM 16-contact lead. **C** X-rays of leads implanted in SCI participants; Left: paddle implanted during T12 laminectomy surgery; Right: paddle implanted after surgery overlying the T12-L1 epidural space. **D** Left: front view; Middle: side view; and Right: back view showing the placement of EMG electrodes in blue. Electrodes were placed on the 1. Right Iliopsoas (R-IL), 2. Right Rectus Femoris (R-RF), 3. Right Tibialis Anterior (R-TA), 4. Right Extensor Hallucis Longus (R-EHL), 5. Right Gastrocnemius (R-G), 6. Left Iliopsoas (L-IL), 7. Left Rectus Femoris (L-RF), 8. Left Tibialis Anterior (L-TA), 9. Left Extensor Hallucis Longus (L-EHL), and 10. Left Gastrocnemius (L-G). **E** Integrated Computer-Nicolet EDX EMG system powered by Viking software used to acquire sEMGs during the BMCA. **F** BMCA tasks performed by the SCI participants and control participants after electrode placement (**G**). **H** Sample sEMGs, which were analyzed to understand changes in neuromuscular control using spinal map analysis, muscle synergy analysis, and fractal analysis (**I**–**K**, respectively)

This study has been approved by the Hennepin Healthcare Research Institute Institutional Review Board with an Investigational Device Exemption from the United States Food and Drug Administration. The study protocol is registered with ClinicalTrials.gov (NCT03026816). We analyzed six participants with motor and sensory complete SCI with AIS A, [21] who completed at least 7 follow-up sessions. The demographic and medical information of each participant is listed in Table 1. The injuries for all SCI participants were between spinal levels T4 and T8. All SCI participants were implanted with an epidural stimulator consisting of a three-column, 16-contact paddle lead through a T12-L1 laminectomy, and an internal pulse generator (IPG) with a primary cell (Tripole and Proclaim Elite, Abbott, Plano, TX, United States) was placed subcutaneously in the lower lumbar area under general anesthesia, as shown in Fig. 1. Follow-up visits were performed monthly for up to one year (13 follow-ups). SCI participants from the ESTAND study that had completed at least 7 of the follow-up sessions were included in this analysis. A detailed description of the study can be found in previous publications [9, 10, 22]. In addition, nine healthy participants (5 males and 4 females) were also recruited to undergo the BMCA as controls for this study. The control participants’ approximate average age was 31 years. At each follow-up session after surgery, SCI participants underwent a BMCA with and without stimulation [9].

**Table 1:** Demographic data

Pat. ID	Age (Decades)	Sex	AIS Score	Injury level (Spinal section)	Time Since Injury (Years)	Sessions
SCI001	30s	F	A	T4	8	13
SCI002	40s	M	A	T8	18	13
SCI003	50s	F	A	T5	6	13
SCI004	60s	M	A	T5	4	13
SCI005	30s	M	A	T4	9	7
SCI006	20s	M	A	T6	2	10

Pat.ID SCI001, SCI002 demonstrated movement control without active stimulation in the last follow-up session

### BMCA protocol

The BMCA task is an electrophysiologic assessment of voluntary motor control that involves relaxation, reinforcement maneuvers (deep breath, neck flexion, Jendrassik maneuver, and bilateral shoulder shrug), and voluntary leg movements (BL hip flexion/extension, isolated hip flexion/extension of left and right side, BL ankle dorsiflexion/plantarflexion followed by isolated dorsiflexion/plantarflexion of left and right foot) [9, 23]. Participants are allowed to flex their knees during the hip flexion activity.

In each trial, a two-toned auditory cue sounded twice to signal the control participants and SCI participants to begin and end the movement. Three trials of each voluntary movement were performed by following the two-tone auditory cue played three times. For example, after hearing the first tone, the participants would flex their hips, after the second tone, the participants would extend their hips. Three trials were performed for each voluntary movement.

For the control participants, the BMCA protocol was conducted once. For the SCI participants, the complete BMCA protocol was conducted twice at each follow-up visit, once with stimulation and again without stimulation. If the SCI participants were not able to perform the movement, they were asked to follow the protocol and perform as they were able. During each follow-up session, three trials of data were acquired with and without stimulation for all six voluntary movements for a total of 36 trials = 2 conditions $\times$ 3 trials $\times$ 6 voluntary movements. For the control participants, we acquired 18 trials = 1 condition $\times$ 3 trials $\times$ 6 voluntary movements.

### Parameter optimization

In the ESTAND study, we programmed the settings of the stimulator one month after surgery. We performed parameter optimization in three different phases. The optimal spatial configuration of electrodes was determined during follow-up sessions 1–3 in the clinic. In these sessions, the configurations were optimized based on maximal movement and EMG signals. Using these optimal spatial configurations, the temporal parameters (frequency and pulse width) were then optimized based on the participant’s preference in later follow-up sessions 4–11.

In each follow-up session, the stimulator was programmed with 15 temporal parameters settings that were to be tested at home. The SCI participants were able to directly control the amplitude of stimulation based on their comfort level. The stimulation amplitude was 8.5 mA on average and ranged from 2.9 to 16 mA. To determine the benefits of stimulation, participants were asked to use one setting throughout the day at home and record movements during an app-driven triple flexion and extension task while wearing accelerometers. At the end of the day, the SCI participants provided an evaluation using an online survey indicating their overall preference between the current setting and the previous day’s setting. This evaluation data was used to develop preference maps using machine learning models. In the last follow-up sessions (12–13), other spatial parameters were tested with the determined optimal temporal parameters as a refinement step. The preferred stimulation frequency was typically in the range of 28–44 Hz, and the preferred pulse width was typically in the range of 400–500 us. A more detailed description of parameter optimization is presented in [11].

### EMG recording, processing, and segmentation

sEMG recordings were acquired during BMCA tasks at a sampling rate of 600 Hz using the Nicollet EDX EMG system for the following 10 lower limb muscles of the right and left sides: iliopsoas (R-IL, L-IL), rectus femoris (R-RF, L-RF), tibialis anterior (R-TA, L-TA), gastrocnemius medial (R-G, L-G), and extensor hallucis longus (R-EHL, L-EHL). Twenty mm disc-shaped bipolar electrodes with an interelectrode distance of 2 cm were placed on the surface of the muscle belly after cleaning the skin with alcohol wipes for sEMG recordings.

MATLAB (2020b, Natik MA) was used to process and analyze the sEMG data. Each channel was filtered using a 6th-order bandpass Butterworth filter from 10 Hz to 300 Hz. For muscles in close proximity to the stimulation electrode and thus having strong stimulus artifacts, such as the R-IL and L-IL, a 5th-order median filter was used to remove the stimulation artifact. Instantaneous power, $sEMG_{\text{RMS}}$, was estimated by calculating the root mean square (RMS) envelope over 100 millisecond non-overlapping windows.

The beginning and end of each movement, as indicated by auditory tone, were labeled with timestamps in the EMG acquisition system. These timestamps were used to segment the voluntary movement $sEMG_{\text{RMS}}$ for each trial. Because each trial was of a different length, the segmented $sEMG_{\text{RMS}}$ signals were time-normalized by interpolation to 7000 time points, resulting in an $sEMG_{\text{RMS}}$ matrix size of 3 trials $\times$ 10 channels $\times$ 7000 time points.

sEMG processing was performed for each voluntary movement (VM = 1 to 6) under the complete BMCA protocol (condition $\times$ trials $\times$ [channel $\times$ time points] $\times$ VM = 1 $\times$ 3 $\times$ [10 $\times$ 7000] $\times$ 6) for control participants and (2$\times 3\times$[10 $\times$ 7000]$\times$6) for SCI participants (with and without stimulation conditions).

### Complexity analysis

We used Higuchi Fractal Dimension (HFD) analysis on the $sEMG_{\text{RMS}}$ to quantify the complexity in the $sEMG_{\text{RMS}}$ amplitude over time [24]. $sEMG_{\text{RMS}}$ was analyzed over time as a sequence of N samples, *E*(1), *E*(2), ....*E*(*N*), and k new time series sequences were created.

1 $$\begin{aligned} E_k^m=E(m),\ E(m+k),\ E(m+2k)......E(m+int[(N-m)/k]k) \end{aligned}$$

In this equation, the initial time (m) = 1,2,3....k, the time interval (k) = 2, 3,.....$k_{\text{max}}$, and int is the integer part of the real number. The length $L_m(k)$ of every time-series sequence was constructed using equation (2).

2 $$\begin{aligned} L_m(k)\ =\frac{1}{k}\ \left[ \left( \sum _{i\ =\ 1}^{int\left[ \frac{N-m}{k}\right] }\left| E(m+ik)-E(m+(i-1)k)\right| \right) \right] \frac{N-1}{int\left[ \frac{N-m}{k}\right] k} \end{aligned}$$

$L_m(k)$ is averaged across all m, resulting in an average curve length of $L_k$, which is computed from Eq. (3). Moreover, the HFD was determined from the slope of the best fit of $ln(L_k)$ vs. $ln(\frac{1}{k})$ using Eq. (4).

3 $$\begin{aligned} L_k= & {} \ \frac{\sum _{m\ =\ 1}^{k}{L_m(k)}}{k} \end{aligned}$$

4 $$\begin{aligned} HFD= & {} \frac{\ ln(L_k)}{ln(\frac{1}{k})}\ \end{aligned}$$

HFD analysis was performed on each muscle during the voluntary movements in the BMCA task. The HFD complexity was estimated with and without stimulation for every voluntary movement and muscle over the follow-up sessions. We compared the HFD complexity of the control participants with that of the SCI participants with and without stimulation. In addition, a non parametric test was performed on the complexity values of the SCI participants’ muscles over the follow-up sessions with and without stimulation.

### Spinal motor output

We also studied the effect of epidural stimulation on spinal cord activity using maps of muscle activity. We mapped the $sEMG_{\text{RMS}}$ onto the estimated rostro-caudal region of the motor neuron (MN) pool in the spinal cord from segments L1 to S1 [14, 15]. A myotomal chart developed by Sharrard and shown in Eq. (5) was used to calculate the maps of putative alpha motor neuron activation [15, 25]. The myotomal chart provides the connection between each muscle and a specific spinal segment. The chart models muscle innervation from the spinal segments via the alpha motor neurons. The activity in the spinal cord was computed from an SCI participant’s voluntary movement with and without stimulation.

5 $$\begin{aligned} S_j,_s\ =\frac{\sum _{\ \ {i}\ =\ {1}}^{{m}}{{k}_{{ij}}{E}_{{i},{s}}}}{{n}_{j}} \end{aligned}$$

Here, $S_{j,s}$ is the estimated spinal motor output from the jth segment with s samples, $E_{i,s}$ is the $sEMG_{\text{RMS}}$ signal from channel/muscle i, m is the number of sEMG signals, $k_{ij}$ is the weighting coefficient of the ith muscle corresponding to the jth segment, and $n_j$ is the number of $sEMG_{\text{RMS}}$ values to the jth segment. The weighting coefficient (${k}_{ij}$) values in the spinal maps are based on those from previous studies [15, 25, 26]. The spinal map developed for our study can be found in Additional file 1: Table S1.

### Muscle synergy extraction

Muscle synergies are considered to be organized in the CNS as low-dimensional muscle coactivation patterns used to form movement [18, 27]. In short, muscle synergies are the functional building blocks of movement extracted from sEMG linear envelopes. Muscle synergies and their activation coefficients are generally estimated using non-negative matrix factorization (NNMF) [12], and they can explain the neurophysiological characteristics of a movement. We used the NNMF algorithm to estimate muscle synergies from the $sEMG_{\text{RMS}}$ [28]. The $sEMG_{\text{RMS}}$ for each segmented trial was amplitude-normalized between 0 and 1, scaling from the minimum to the maximum recorded value. The segmented trials within each voluntary movement were ensemble-averaged, forming an $sEMG_{\text{RMS}}$ matrix for each volitional movement of size (Muscles $\times$ timepoints = 10 $\times$ 7000). The NNMF algorithm was applied to this matrix to estimate muscle synergies. A mathematical model for time-invariant muscle synergies is given by equation (6).

6 $$\begin{aligned} E_{m\times {n}}^k= \sum _{{i}\ ={1}}^{k}{W}_{{m}\times {i}}{\cdot {{A}}}_{{i}\times {n}} \end{aligned}$$

Here, $E^k$ is the $sEMG_{\text{RMS}}$ signal reconstructed with k extracted synergies, m is the number of muscles/channels, n is the number of samples/time points, W is the synergy (spatial structure), and A is the activation coefficient (temporal structure).

NNMF uses the multiplicative update rule method developed by [28]. Hence, for synergy extraction, it was run 100 times to avoid local optima. Moreover, determining the number of synergies, k, is not trivial [12, 29]; therefore, prior to synergy extraction, we first determined the number of factors/synergies that explain 85$\%$ or more of the total variance, as calculated in Eq. (7).

7 $$\begin{aligned} R_k^2=\ 1-\frac{\sum _{i=1}^{m}\sum _{s=1}^{n}(E_{i,s}\ -\ E_{i,{s}}^k)^2}{\sum _{i=1}^{m}\sum _{s=1}^{n}(E_{i,s}^k-\ {{\widehat{\ E^k}}_i})^2} \end{aligned}$$

Here, $E_{i,s}$ is the actual $sEMG_{\text{RMS}}$ signal, $E_{i,s}^k$ is the reconstructed $sEMG_{\text{RMS}}$, $R_k^2$ is the total variance explained by the first k components, and ${\hat{E}}$ is the mean of the reconstructed $sEMG_{\text{RMS}}$.

The number of factors defined in the NNMF was selected to range from 1 to 10, where 10 is the maximum number of synergies that can be extracted, as determined by the number of muscle groups recorded. The $sEMG_{\text{RMS}}$ signals were reconstructed for each NNMF factor (1-N), and the $R_k^2$ value for each defined synergy/factor was computed and plotted using Eq. (7). Based on previous studies, we defined k as the value for which $R_k^2$ meets a minimum threshold of 85$\%$ [30].

The muscle synergies were extracted for SCI participants’ voluntary movements during stimulation and without stimulation over the follow-up sessions. The control participants’ muscle synergies were also extracted during their single visit. The control participants’ muscle synergies were used to understand how muscle synergies change over follow-up sessions with stimulation, as we have previously observed that long-term eSCS improves volitional movement [9].

### Comparison of muscle synergies and their activation coefficients

To understand the impact of stimulation on muscle synergies, we compared the $R^2$ curves and structures of muscle synergies (muscle loadings within the synergy vector) with and without stimulation during the BMCA. The $R^2$ curves for control participants and SCI participants were compared for each voluntary movement performed during the BMCA. Moreover, the effects of stimulation on the $R^2$ curves and muscle synergy structures over the follow-up sessions were also studied.

After extraction of the muscle synergies, the synergies were compared, sorted, and reordered based on similarity. We used the cosine similarity Eq. (8) to compare the muscle synergy structures. An R value close to 1 is considered highly similar, whereas an R value close to 0 suggests independence. Correlations between synergies across all participants were computed. The participant whose synergies had the highest correlation to those of the other participants was used as the template. All other participants’ synergies were then ordered to best match the template synergies for further analysis.

8 $$\begin{aligned} R_{a,b}\ =cos(\vartheta )\ =\ \frac{W_a^T.\ W_b^{SCI}}{||\ W_a^T\ ||\ \times \ ||{\ W}_b^{SCI}\ ||} \end{aligned}$$

Here, $W_a^T$ is the template synergy and $W_b^{SCI}$ is the synergy for all other SCI participants compared to the template. a and b (1,2,...k,) are the number of synergies compared for SCI participants. The same procedure was implemented to organize the synergies of the control participants.

To compare the activation coefficients of respective muscle synergies, a zero-lag cross-correlation was used. A cross-correlation value close to 0 suggests a weak correlation, and a cross-correlation value close to 1 or $-1$ suggests a strong positive or negative correlation, respectively.

### Statistical analysis

To determine if the data were normally distributed, a Kolmogorov-Smirnov test was used. We used a Wilcoxon Sign Rank test and a Mann–Whitney U test as nonparametric tests. The Wilcoxon Sign Rank test was used to compare the effect of stimulation on SCI participants. The Mann–Whitney U test was used to compare the muscle activation complexity between controls and SCI participants. We also used the Independent Groups Equivalence test to compare the equivalence of the $R^2$ values explained by four synergies in SCI participants and control participants at the end of the study. For statistical analysis, p < 0.05 was considered statistically significant.

Violin plots were used to display the differences between the mean, the median value of the control participants, and the SCI participants’ muscle activities with and without stimulation over the follow-up sessions.

## Results

### Epidural stimulation restores independent muscle activity

We first studied the effect of stimulation on independent muscle activity during BMCA tasks. Visual inspection of the sEMG waveforms indicated the restoration of muscle activity in the SCI participants during eSCS. However, compared to the control participants, their sEMG waveforms were not as strong or concise in time, and they were localized to the specific muscle groups required to complete the task.

**Fig. 2:**
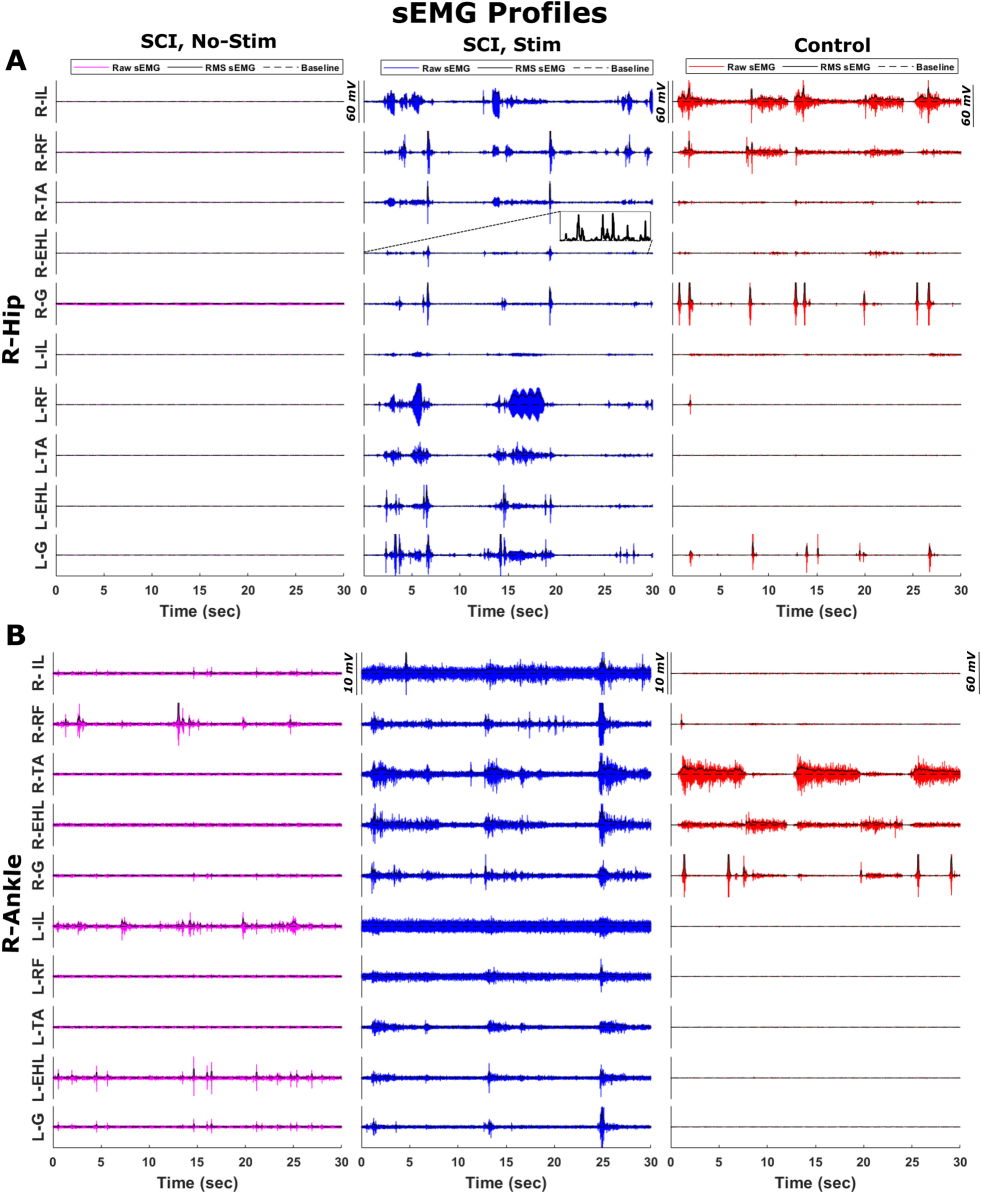
Raw sEMG profiles and their RMS envelopes obtained during BMCA tasks for an SCI participant and a control participant. Examples of sEMG profiles obtained during BMCA tasks involving **A** right hip and **B** right ankle movement by an SCI participant without stimulation (left column), SCI participant with stimulation (middle column), and control participant (right column). An RMS envelope calculated over 100 ms is indicated by a solid black line. Inset shows magnified RMS

Figure 2 shows example sEMG profiles for a control participant and an SCI participant performing unilateral hip and ankle movements (right hip flexion/extension and right ankle plantar/dorsiflexion). During unilateral movement, the SCI participant showed increased sEMG amplitude on not only the ipsilateral side due to stimulation but also for the majority of the muscle groups on the contralateral side. In the control participants, only the muscles directly involved in the task showed a higher sEMG amplitude. During the BMCA tasks, we found that stimulation evoked increased muscle activity with some asymmetry in the SCI participants. This asymmetric activation was likely a result of the involuntary contraction of muscles on the contralateral side.

### Muscle activation profile complexity

**Fig. 3:**
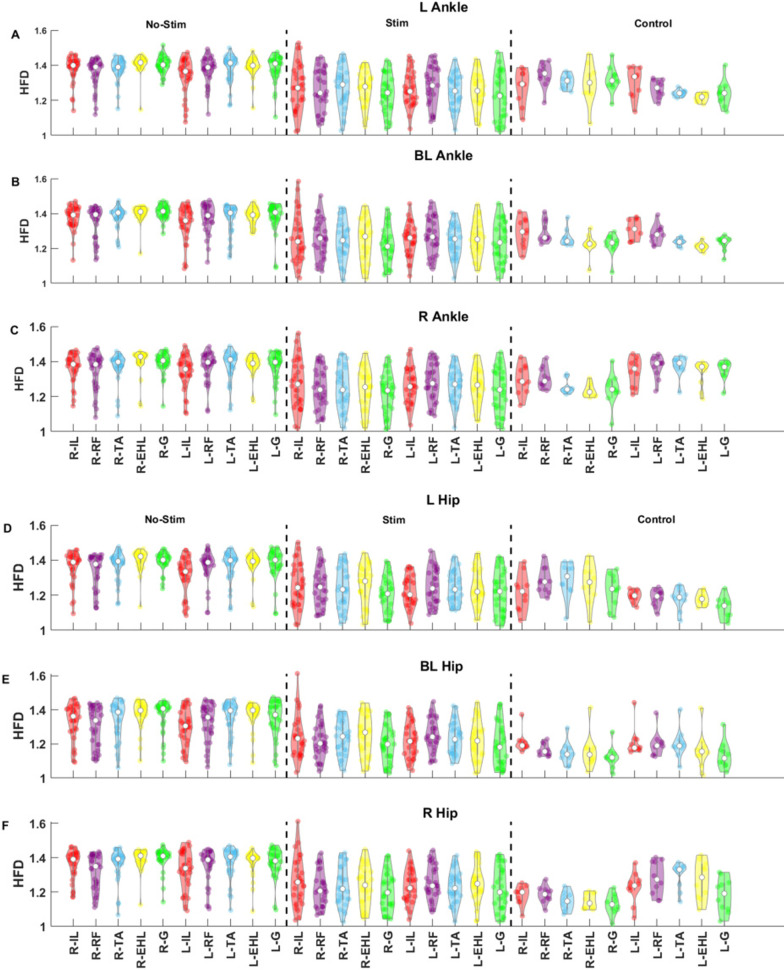
Higuchi Fractal Dimension (HFD) plots of sEMGs during BMCA tasks. HFD is used to estimate the complexity of $sEMG_{\text{RMS}}$ signals. The HFD data points in violin plots represent follow-up sessions 7–13 of all SCI participants. The top panels (A, B, C) display ankle movement during the BMCA task, and the bottom panels (D, E, F) correspond to hip movement. The top row in each panel (A, D) represents left ankle and hip movements, the second row (B, E) represents BL ankle and hip movements, and the bottom row (C, F) represents right ankle and hip movements. Within each row, the HFD is displayed for SCI participants without stimulation (left), SCI participants with stimulation (middle), and control participants (right). The control participants have much lower complexity than the SCI participants without stimulation. With stimulation of the SCI participants, the complexity decreases. In the control participants, the complexity is lower in the muscles involved in the task than in the muscles on the contralateral side

We also studied the effect of stimulation on the complexity of the $sEMG_{\text{RMS}}$. The complexity of muscle movement is estimated using HFD, which detects the patterns and smoothness of muscle movement [31]. Figure 3 shows violin plots of the fractal dimensions for control participants and for SCI participants with and without stimulation over the follow-up sessions. In SCI participants with stimulation, the HFD of muscle activity during the BMCA tasks was reduced significantly (p < 0.05, Wilcoxon Sign Rank test) compared to that of SCI participants without stimulation. With stimulation, the HFD median values for muscle activation were close to those of the control participants, as the complexity between control and SCI participants was statistically insignificant (p > 0.05, Mann–Whitney U test), as shown in Fig. 3. To set the overall significance threshold at $\alpha =0.05$ for significant differences in complexity between SCI and control participants over the 67 statistical tests evaluated, we used a *post hoc* Bonferroni correction with p = 0.0007. The estimated p-values for the control participants vs the SCI participants with stimulation and for the SCI participants with and without stimulation are shown in Table 2. The lower HFD values across SCI participants during stimulation compared to without stimulation suggest that the complexity of muscle activation decreases as a result of eSCS. Moreover, unlike in the control participants, the reduced complexity of muscle activation for the SCI participants with stimulation was not dependent on the task. In the control participants, muscles involved directly with the task had lower fractal dimensions than those not directly involved, indicating that selective activation of these muscles reduces the complexity.

**Table 2:** Stimulation effect on the complexity of $sEMG_{\text{RMS}}$

Control vs Stim						
Muscle	BMCA Tasks					
Name	BL-Hip	R-Hip	L-Hip	BL-Ankle	R-Ankle	L-Ankle
R-IL	0.42	0.1	0.42	0.35	0.56	0.69
R-RF	0.08	0.44	0.29	0.29	0.25	0.06
R-TA	0.01	0.02	0.26	0.81	0.81	0.46
R-EHL	0.02	0.03	0.86	0.52	0.58	0.40
R-GAS	0.03	0.07	0.48	0.69	0.63	0.04
L-IL	0.72	0.93	0.46	0.04	0.03	0.19
L-RF	0.06	0.48	0.10	0.72	0.02	0.58
L-TA	0.40	0.05	0.12	0.79	0.001	0.71
L-EHL	0.13	0.79	0.05	0.15	0.04	0.19
L-GAS	0.26	0.41	0.06	0.88	0.006	0.72

No stim vs Stim						
Muscle	BMCA Tasks					
Name	BL-Hip	R-Hip	L-Hip	BL-Ankle	R-Ankle	L-Ankle
R-IL	**0.00003**	**0.00005**	** 0.00004**	**0.0001**	**0.0001**	**0.00021**
R-RF	**0.0005**	**0.00001**	**0.00024**	**0.00015**	**0.0001**	**0.00025**
R-TA	**0.00001**	**0.0001**	**0.0005**	**0.00002**	**0.0001**	**0.00001**
R-EHL	** 0.0001**	** 0.0002**	**0.00001**	**0.00002**	**0.00008**	**0.00002**
R-GAS	**0.00001**	**0.0001**	**0.00005**	**0.0002**	**0.0002**	**0.00002**
L-IL	**0.0002**	**0.0001**	**0.00003**	**0.0005**	**0.00014**	**0.0001**
L-RF	** 0.0002**	**0.0005**	**0.00005**	**0.0006**	**0.0005**	**0.00008**
L-TA	**0.0004**	**0.00005**	**0.00001**	**0.0003**	**0.0001**	**0.00003**
L-EHL	**0.0003**	**0.0004**	**0.00001**	**0.0001**	**0.0004**	**0.0001**
L-GAS	**0.0005**	**0.0004**	**0.00002**	**0.0004**	**0.00005**	**0.00001**

The p values shown in the table are based on Wilcoxon sign rank (No stim vs Stim) and Mann–Whitney U tests (Control vs Stim). The bold fonts within the table indicate statistically significant differences p < 0.0007

### Estimated spinal motor neuron activity based on muscle activation

**Fig. 4:**
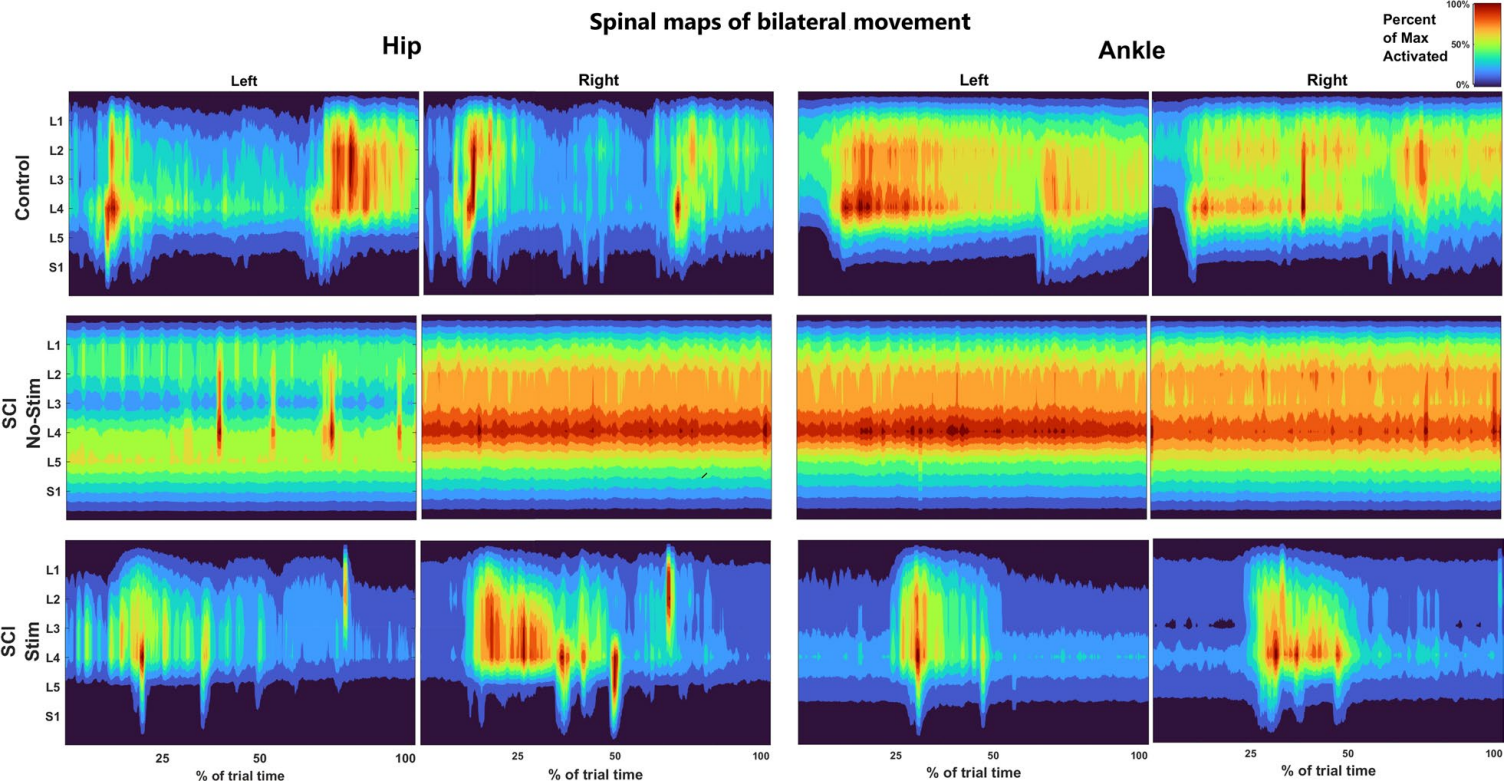
Spinal activity mapped during BL hip and BL ankle movements during BMCA in SCI participants and control participants. The x axis corresponds to movement as a percentage of task completion (temporal domain); the y axis depicts the spinal segment in the rostro-caudal plane from L1-S1 (spatial domain); and the heatmap represents the estimated alpha motor neuron activity. These estimated spinal activation patterns were obtained by mapping each muscle activation onto the relevant spinal segment based on the Sharrard chart [25]. The left columns are the spinal activation patterns generated from muscle activity during BL hip movement. The right columns are the spinal activation patterns generated from muscle activity during BL ankle movement. The spinal activation patterns reveal that the estimated spinal activity is more localized in the temporal domain under stimulation conditions than in the control. The localization of spinal activity during BL hip movement suggests improper hip extension, as motor neurons are active mostly in the first half of contraction. The observed localization during BL ankle movement suggests reduced control as the tibialis anterior and gastrocnemius are co-contracted

We then studied the effect of epidural stimulation on spinal cord activity by mapping the $sEMG_{\text{RMS}}$ on the rostral-caudal plane of the spinal cord during BMCA tasks. This mapped spinal activity provided an estimate of alpha motor neuron activity. We first compared the mapped spinal activity (averaged over all trials) of each participant with and without stimulation and later compared the activity with those of the control participants. Figure 4 shows the averaged mapped spinal activities of the control and SCI participants with and without stimulation during BL movements. The estimated alpha motor neuron activity amplitude from each segment was significantly different between no stimulation and stimulation conditions in the SCI participants (p < 0.01, Wilcoxon sign rank). In addition, Fig. 4 depicts localized estimated motor neuron activity in the temporal domain due to stimulation, suggesting that the mapped spinal activity is sensitive to epidural stimulation in the temporal domain.

The difference between the control and SCI participants in estimated alpha motor neuron activity amplitude for each segment is also statistically significant (p < 0.001, Mann Whitney U test). From Table 2 in the supplementary material, most SCI participants showed localized motor neuron activity in a single phase (either at the beginning or the end of the trial) during BMCA tasks. On the other hand, the controls exhibited clearly separated activation events during the flexion and extension phases of the trial, as shown in Fig. 4. In Fig. 4, the controls do not show the clear localization of estimated motor neuron activity during ankle movement that was seen with the SCI participants. Hence, the estimated alpha motor neuron activity of SCI participants with stimulation is more localized in the temporal domain than is the estimated alpha motor neuron activity of control participants.

### Muscle coordination improves with eSCS therapy

**Fig. 5:**
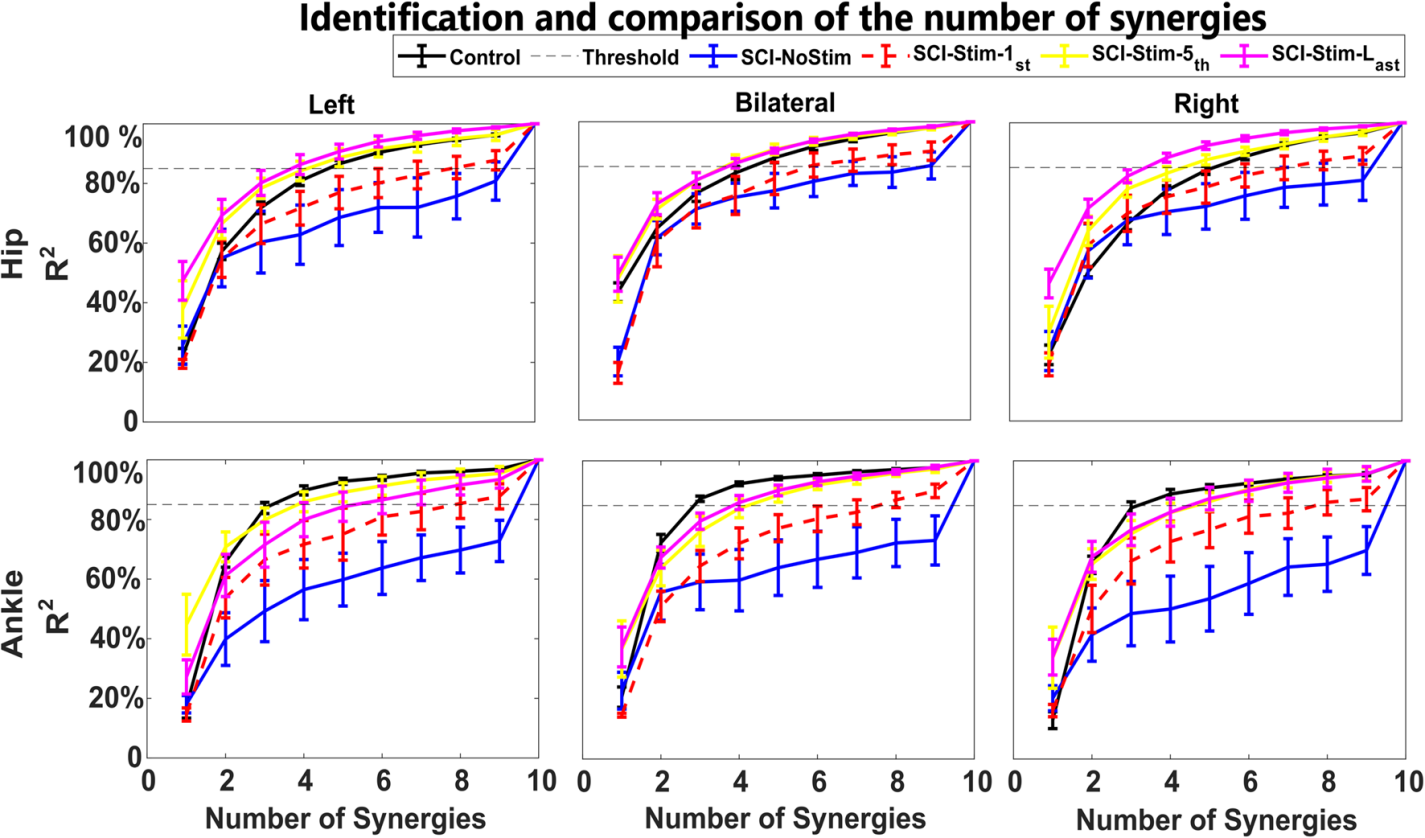
Number of muscle synergies determined from coefficient of determination ($R^2$) curves across different therapeutic conditions. The number of synergies is determined from a threshold of 85$\%$ of the $R^2$ value. The $R^2$ value (y axis) is plotted against the number of factors/synergies (x-axis) for SCI participants under different conditions (no stimulation and stimulation in the 1st session, 5th session, and last session) and control participants. The left, middle, and right panels in the top row display left, BL, and right hip movements. The left, middle, and right panels in the bottom row display left, BL, and right ankle movements. In the absence of stimulation, all ten components were required to explain at least 85$\%$ variability in the data. During eSCS, a dose-dependent reduction in dimensionality was observed. In the last session, the number of synergies for the SCI participants was very close to that for the control participants. We found that a total of four muscle synergies were needed to explain 85% of the variance in the data-the same number of synergies needed for the control participants. Moreover, two SCI participants (SCI001, SCI002) were able to maintain the reduced dimensionality and improved motor function in the absence of stimulation. The $R^2$ curve for them is shown in Additional file 1: Figure S2

We observed that the coordination capabilities of muscle groups during movement significantly improved with stimulation and over time, as measured by a decrease in the number of synergies. Figure 5 shows the $R^2$ curves of SCI participants with and without stimulation and of the control subjects. The number of muscle synergies for the last session and the control participants was statistically equivalent (Additional file 1: Figure S1). The slopes of the $R^2$ curves increase faster for hip movement than for ankle movement with stimulation over the follow-up sessions ($1^{st}$, $5^{th}$, and last sessions), suggesting the earlier restoration of the muscle synergies associated with hip movement than with ankle movement.

**Fig. 6:**
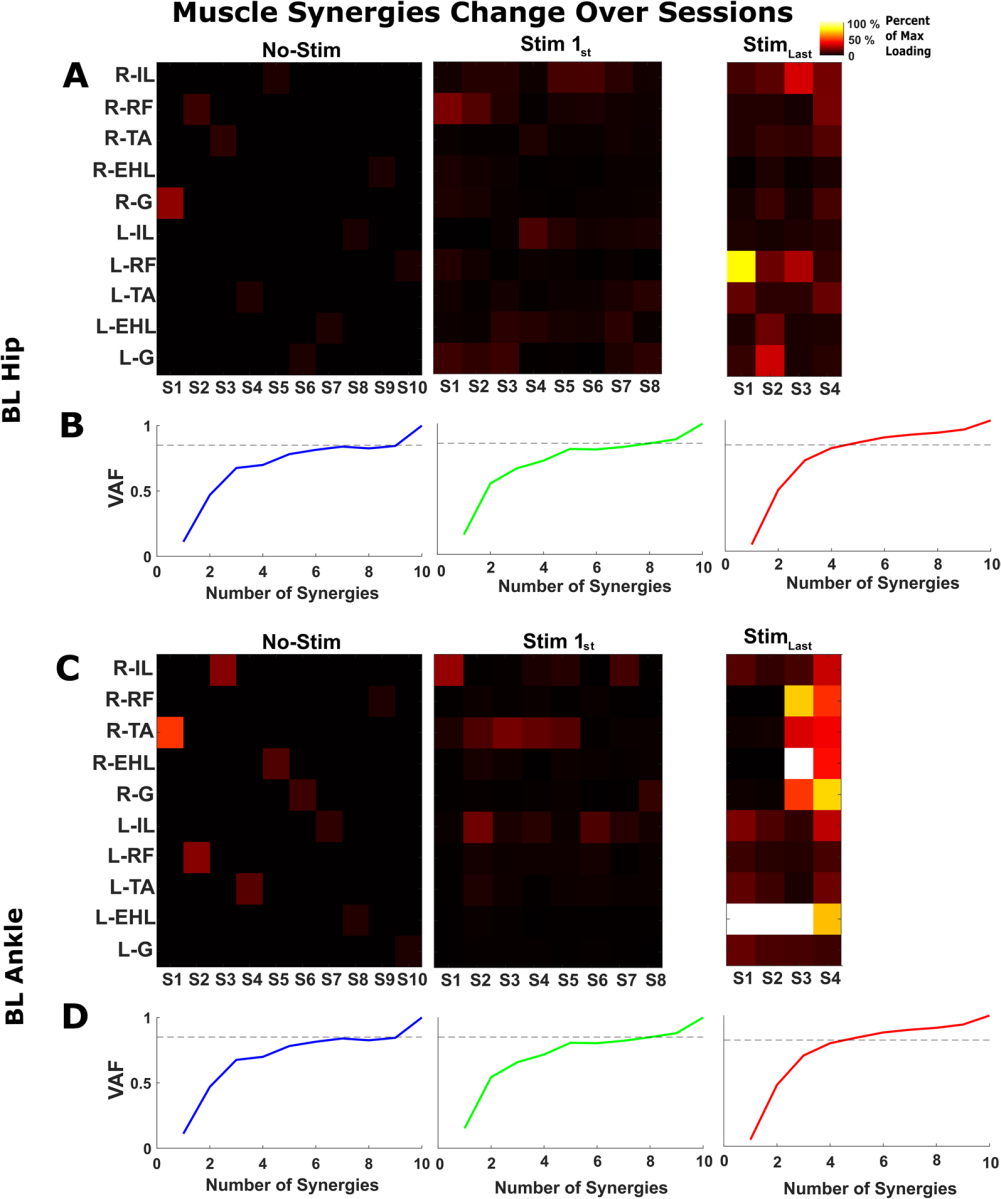
Spatial and temporal changes in muscle synergies. **A**,** C** Plots of muscle loading in which the x-axis displays the synergies (S1, S2, S3—Sn) and the y-axis displays the muscle groups. Each pixel in **A**,** C** is a muscle contribution/loading value within a synergy. **B**, **D** $R^2$ curves; the dashed line is 85$\%$ of the total variance and is used as a threshold value for identifying the number of muscle synergies. We extracted ten, eight, and four muscle synergies based on the $R^2$ threshold value across SCI participants without stimulation and with stimulation (1st and last session). Besides differences in the number of synergies, the muscle synergy structures (muscle loading values in a factor) of BL movements also changed between the first and last stimulation session. Relative to the first day of stimulation, the last day exhibited higher muscle loadings within a synergy and a smaller synergy space. Thus, both the structure and number of muscle synergies changed with eSCS

Figure 6 shows the muscle loadings for the extracted synergies up to the number of synergies for no stimulation, the first session with stimulation, and the final session with stimulation during BL hip and ankle movement tasks. As the number of synergies decreased, the structure of the muscle synergies also changed, as indicated by changes in the muscle loading values within a synergy during no stimulation, the first session with stimulation, and the final session with stimulation. Without stimulation, each muscle loading represented an individual muscle, indicating that the activation of each muscle was independent, and thus, no synergies were found. In the first session with stimulation, the muscle loadings increased, and few synergies were observed, where multiple muscles had significant loadings in the same synergy. In the final session, the muscle loadings were much stronger, and the synergies showed coordination between several muscles, with several muscles having high loadings within the same synergy and only 4 synergies being needed to explain 85% of the variance. These synergy loadings support our hypothesis that epidural stimulation restores muscle synergies by modulating both the structure and the number of synergies.

### Muscle synergies shared across SCI participants

Based on the ordering scheme described in the methods, we calculated the R values across SCI participants for each BMCA task and ordered similar muscle synergies. The synergies extracted for SCI participants (last session) and control participants were compared, as the synergies in the last session exhibited dimensions/numbers consistent with those of the control participants.

**Fig. 7:**
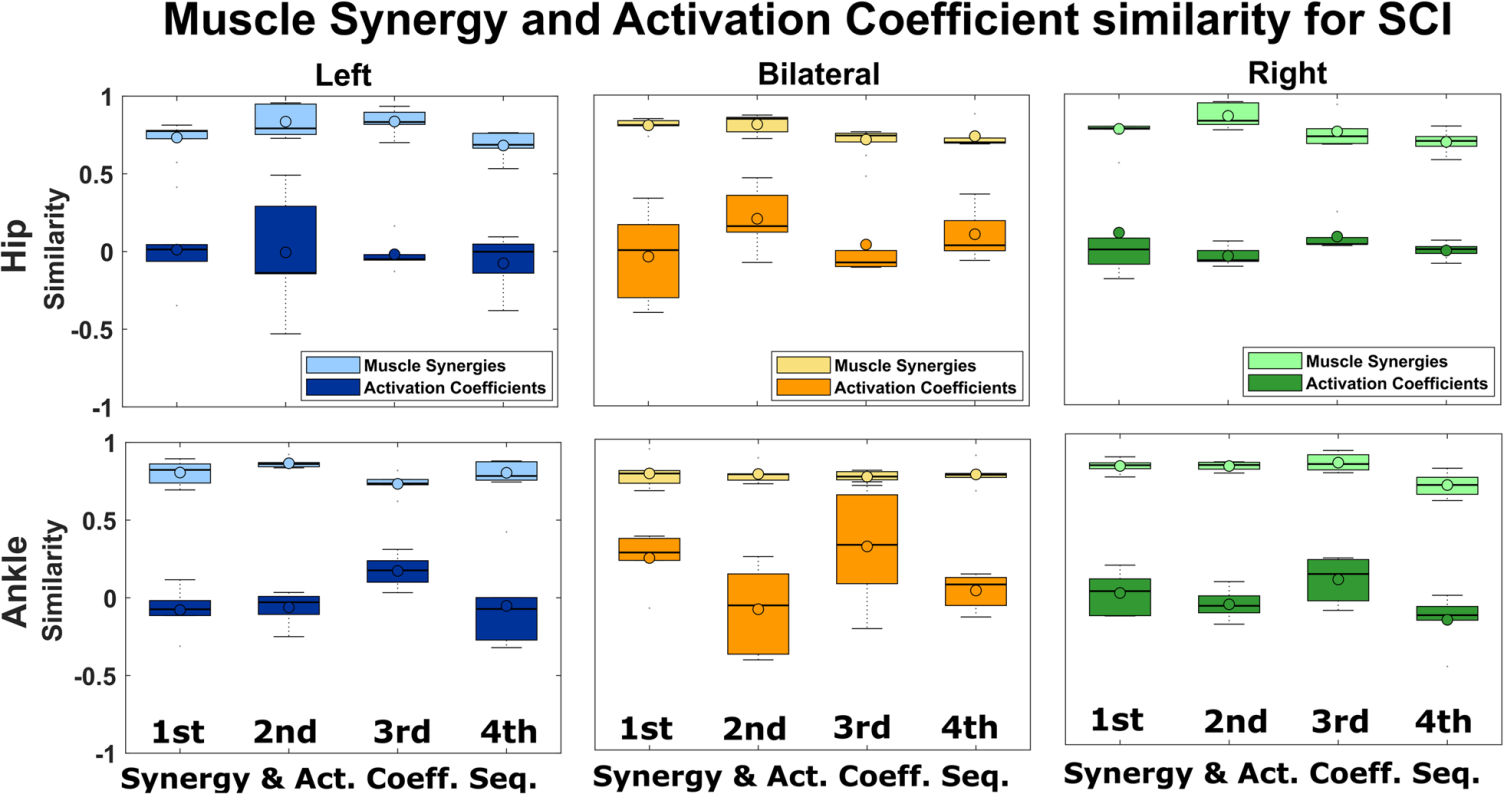
Comparison of synergies and activation coefficients across SCI participants. We observed four muscle synergies that were similar across SCI participants with epidural stimulation. The R values are plotted for the synergies and their activation coefficients across SCI participants. In the top row, the left, middle, and right panels display left, BL, and right hip movements, respectively. In the bottom row, the left, middle, and right panels show left, BL, and right ankle movements, respectively. The R values were computed using cosine similarity for the muscle synergies and zero-lag cross-correlation for the activation coefficients. Muscle synergies are more consistent than the respective activation coefficients across SCI participants

We found that the first four synergies were similar across SCI participants for each BMCA task, as shown by the high correlation (R values) depicted in Fig. 7. This result indicates that the coordination of flexors and extensors improved. However, temporal activation, which is likely determined by cortical control [32], is not directly affected by stimulation, and so the temporal patterns were not restored, as evidenced by weak correlations (R values near zero). In deafferented animal models, synergies are preserved but temporal patterns are weakened [19, 32], suggesting the central organization of synergies with a neural source [12, 33]. Therefore, the restoration of similar synergies but not the temporal patterns across SCI participants with stimulation supports the hypothesis that these synergies originate from a neural source and not a task source.

### Muscle synergies of SCI participants vs control participants

**Fig. 8:**
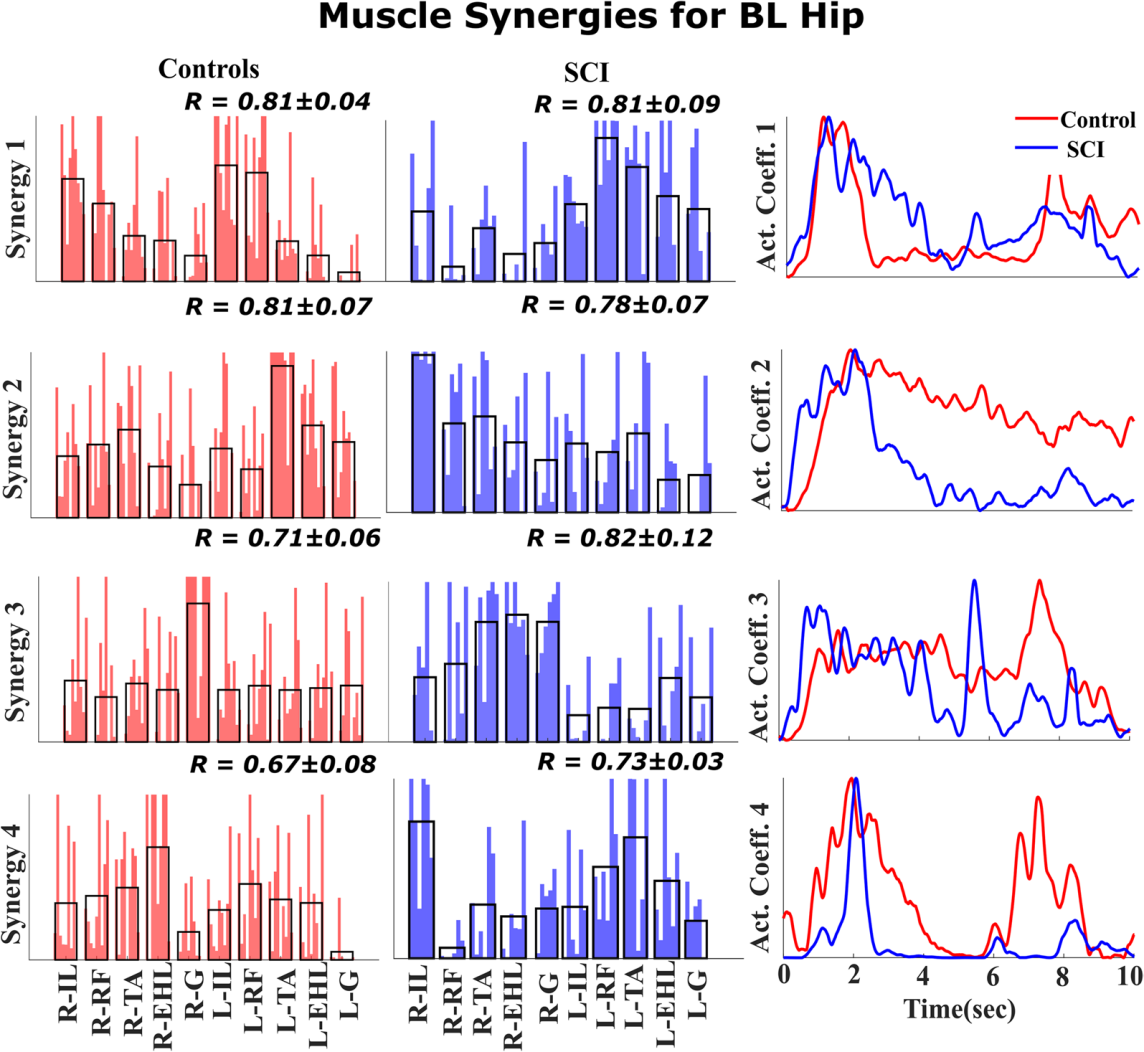
Muscle synergies for BL hip movement Four similar synergies were identified across the control participants (left column) and SCI participants (middle column) during BL hip flexion. The weighting for each muscle synergy is shown for SCI participants and control participants. The average across weightings is shown as a black overlapping bar. In the right column, the activation coefficient is displayed as an average across SCI participants and control participants. Comparison of the BL hip movement synergies of SCI participants with those of control participants reveals asymmetry in the hip flexor muscle activity. The activity of the hip flexor muscles of the left and right sides is split into two separate synergies, Synergy 1 and 2, while in the control participants, Synergy 1 includes activity for both sides

**Fig. 9:**
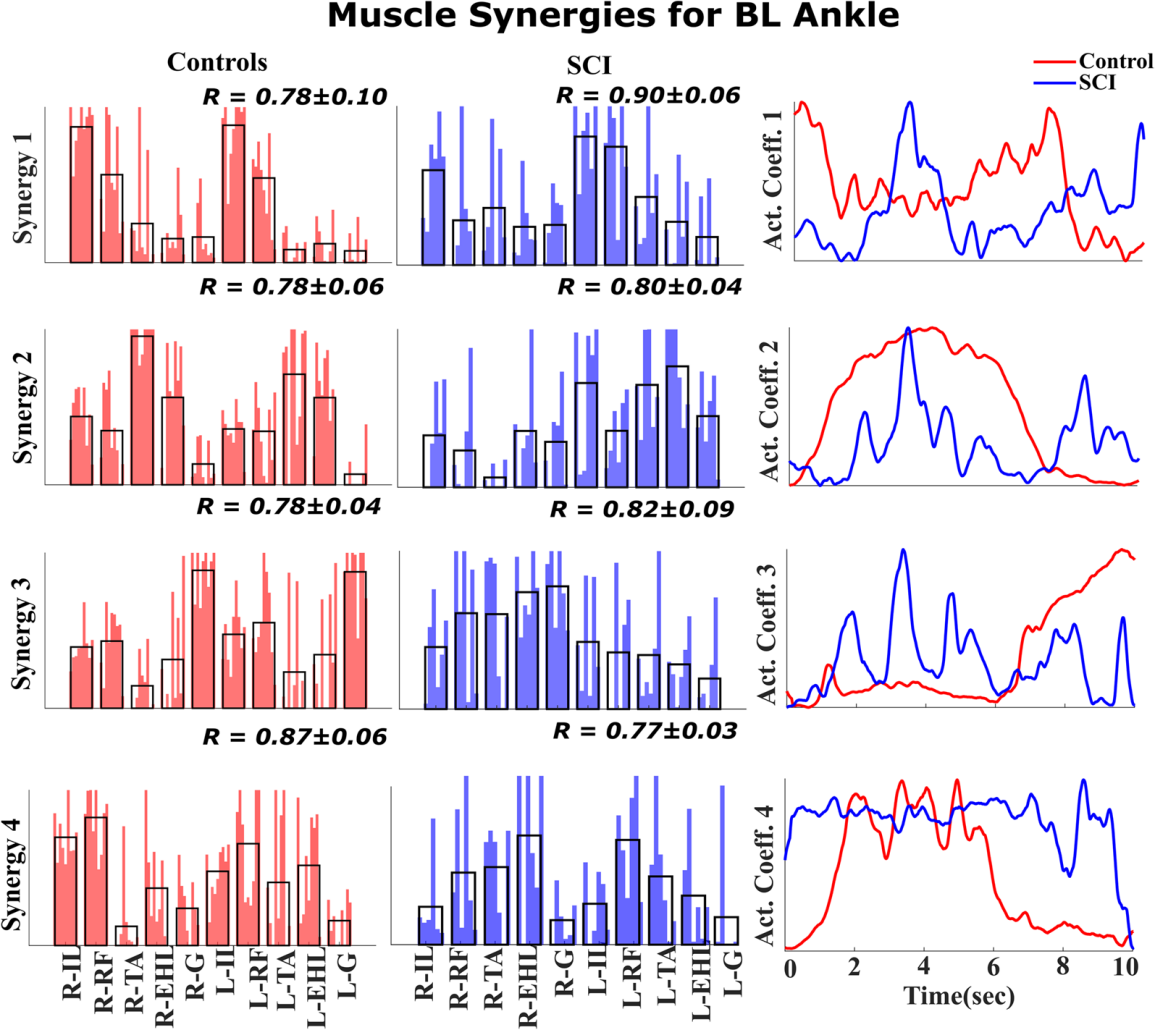
Muscle Synergies for BL ankle movement. Similar to the previous figure, synergies associated with BL ankle movement are plotted across control participants and SCI participants. The muscle synergies for BL ankle movement of the SCI participants also revealed asymmetric muscle contributions. The left- and right-side plantar flexor and dorsiflexor muscles are isolated in two synergies, 2 and 3, whereas for the control participants, a single synergy contains contributions from both sides’ dorsiflexor and plantar flexor muscles

The distinctively higher muscle loading (muscle loading > 0.5) values observed within a synergy among SCI participants and control participants [34] display specific biomechanical functions. Therefore, we compared the muscle loadings of SCI participants with those of control participants. Figure 8 and Fig. 9 show the muscle synergies of SCI participants and control participants.

The first synergy observed during BL hip movement in the control participants was different from that observed in the SCI participants, as seen in Fig. 8. In the control participants, the loadings in Synergy 1 were balanced between the left and right sides, indicating synergy between the two legs during BL movement. However, in the SCI participants, Synergy 1 represented left leg muscles, and Synergy 2 represented right muscles, indicating a lack of synchronization between the legs during BL movement. Moreover, the muscle loadings in the remaining synergies of the SCI participants (Synergy 3 and Synergy 4) and control participants (Synergy 2 and Synergy 3) predominantly represented distal muscles, indicating that they may be associated with supporting knee flexion and foot inversion during BL hip flexion.

We found that the muscle synergies for BL ankle movement in SCI participants were also asymmetric. In the control participants, the muscle loadings associated with left and right plantar flexion are seen in Synergy 2, and those of left and right dorsiflexion are seen in Synergy 3, as shown in Fig. 9. In the SCI participants, the synergies are asymmetric between the left and right sides, as seen in Synergy 2 and 3. Additionally, in the SCI participants, Synergy 1 is more consistent with isometric hip flexion, and Synergy 4 is prominently associated with right foot inversion.

## Discussion

We studied the effect of eSCS on neuromuscular control in participants with motor and sensory complete, chronic SCI. We examined sEMGs acquired from the lower limbs of SCI participants while they performed a motor control task (BMCA) with and without stimulation over several follow-up visits and compared the results with sEMGs acquired from healthy participants. With stimulation, we observed a decreased complexity in muscle activation profiles over time in the follow-up visits. In addition, during the flexion or extension phase of the BMCA tasks, SCI participants with stimulation exhibited more localized motor neuron activity than the control participants, as inferred from their muscle activation. We also studied the muscle synergies of the SCI participants to understand the modulation of neuromuscular control through stimulation in several follow-up sessions; over time, the muscle synergies showed dimensional and spatio-temporal changes. In particular, from the first to the last session, the number of muscle synergies decreased, and the muscle loadings within the synergies increased. In comparison to the control participants, the muscle loadings of the SCI participants were asymmetric between the left and right sides. Overall, our results suggest that epidural stimulation modulates the local spinal circuitry to restore muscle synergies. In the final follow-up visit, the number of synergies of the SCI participants under stimulation matched that of the controls. However, these muscle synergies, while improved in terms of dimensionality, remained significantly different from those of the control participants.

### Epidural stimulation and motor control

eSCS has been shown to restore some volitional control in patients with motor and sensory complete SCI. It is hypothesized that epidural stimulation activates local sensory afferents and motor efferents, which modulates the balance of excitation and inhibition in the spinal cord to restore a dynamic state that is responsive to the remaining supraspinal signals, thereby restoring volitional and autonomic control [10, 35]. Several studies have reported that epidural stimulation in conjunction with simple activity-based therapies restores voluntary control of movement [35]. The therapies range from static postures, such as standing with full weight bearing, to simple dynamic movements, such as assisted stepping [36]. Moreover, epidural stimulation after incomplete SCI has shown restored volitional movement control for complex dynamic tasks such as treadmill walking [37–39]. Recent studies have suggested that the epidural stimulation parameters can be optimized further for in-the-loop [11], closed-loop, and/or phasic stimulation [40] to achieve more efficient voluntary control of movement.

In previous studies, we have shown that eSCS results in increased sEMG amplitudes and restored movement [9, 10]. In this study, we further show that stimulation changes the complexity of muscle activation and activity patterns in the spinal cord. Spinal cord activity was estimated from muscle activity through a mapping procedure to estimate the alpha motor neuron activity in the dorsal roots [15]. The complexity of the movements was estimated through HFD analysis of muscle activity. The mapped spinal activity and complexity analysis performed in this study identified differences in the control of movement between SCI participants and control participants. The mapped spinal activity of SCI participants indicated co-contraction of the distal muscles during BL movement and involuntary contraction of contralateral muscles during unilateral movement.

Mapped spinal activity can be used to identify different phases of movement and has been previously used to identify specific phases of gait [13, 15]. During hip movement by the control participants, the mapped spinal activity had distinct flexion and extension phases. However, for the SCI participants, we observed only a single phase at the onset of movement, representing either flexion or extension. Moreover, during ankle movement, the mapped activity of the SCI participants was not as broadly distributed as that of the control participants as a result of co-contraction of the distal lower limb muscles.

The complexity of all muscle activation in the presence of stimulation, estimated using HFD, decreased during BL movement. Furthermore, the $sEMG_{\mathrm{{RMS}}}$ complexity of the SCI participants was close to that of the control participants during the BL movement. This result suggests that a lower complexity in the sEMG amplitude under stimulation is indicative of the completion of BL BMCA tasks. Contrary to the control participants, the SCI participants showed a decrease in muscle activation complexity on both sides during unilateral movement. This result was caused by the involuntary contraction of the contralateral muscles that occurred during stimulation. The decreased complexity on the contralateral side made it harder to distinguish the differences between the muscle activation complexities of the ipsilateral and contralateral sides. Therefore, the similarity in HFD complexity between the contralateral and ipsilateral sides during unilateral movement could be used as a measure to quantify an SCI participant’s lack of precise motor control. These results also suggest that the lower complexity of sEMG while accomplishing BMCA tasks in SCI participants is dependent on the stimulation parameters rather than the task dynamics.

During volitional movement in SCI participants with stimulation, a large activation of muscle activity was measured with sEMG, but the movement was not necessarily smooth due to a lack of coordination. Clear isolation of the agonistic and antagonistic muscle activations during extension and flexion can be seen in the bottom right panels of Fig. 2, which depict right ankle movement in control participants. In SCI participants, even on their final visit with stimulation (shown in the middle bottom panels), the antagonistic muscles are coactivated together with the agonistic muscles, which results in erratic joint movement. Furthermore, the contralateral muscles are activated involuntarily, showing poor isolation of muscle activation between the left and right sides. Therefore, our future studies will focus on identifying epidural stimulation parameters that improve control to generate more precise limb biomechanics.

### Neural basis of muscle synergies

There is an ongoing debate about whether muscle synergies explain neural changes or task-related changes. In individuals with neurological conditions such as stroke, cerebral palsy, and SCI, the number and structure of muscle synergies changed with the repetition of a single task. Although this result supports the neural basis of synergies, this evidence is correlational [12, 18]. Moreover, in individuals without movement disorders, the muscle synergies remained similar when the same tasks were repeated and changed only when the task changed, supporting the task-related hypothesis [41]. Studies in intact participants have been unable to resolve the competition between these two hypotheses because the afferent drive through intact ascending tracts is modulated when the task is changed; therefore, there is always a neural component to task-based synergies that cannot be isolated [17, 41–43]. However, in the current study, the measurement of changes in muscle synergies under epidural stimulation without the restoration of sensory feedback allows us to isolate the neural effects from the task effects in muscle synergies.

Previous studies have used spinal transection and stimulation in animals to test a direct relationship between changes in neural control and muscle synergies [27, 44–46]. Our study is the first to show changes in muscle synergies over time with neuromodulation in SCI participants, as we tested changes in neural drive directly through stimulation. Therefore, our study adds further support for the neural basis of muscle synergy in humans.

Our results strengthen the evidence for the neural basis of muscle synergies in two ways. First, the high numbers of synergies in SCI participants in the absence of stimulation during BMCA tasks are immediately reduced upon stimulation. This reduction in the number of muscle synergies in the absence of a neural controller is counter to the results of [17] that suggested a non-neural origin of muscle synergies [17]. If synergies were task-related and not neurally controlled, restoring volitional control would not be expected to reduce the number of synergies. The second line of evidence in support of the neural basis of muscle synergies comes from the longitudinal changes observed in this study. A confounding problem in testing the origins of muscle synergies is the adaptation of the CNS to different tasks that reshapes the number and structure of muscle synergies via sensory feedback [42, 43, 47]. However, in this study, we were able to measure changes in synergy in the same task performed during the recovery of participants without the restoration of sensory information to the supraspinal region, as most participants were not able to perform the BMCA task without stimulation. This finding suggests that motor learning-based changes in synergies were precluded; however, it is possible, though unlikely, that the changes result from visual-motor learning. Over the follow-up visits, we observed a decrease in the number of synergies, indicating an improvement in muscle synergy due to neural control that was independent of task control. This finding is consistent with those of previous studies showing changes in muscle synergies due to neural changes in development [18, 48, 49].

### The number or dimensionality of synergies and efficient movement control

Our results show that long-term stimulation impacts the dimensionality/number of muscle synergies. However, the number of muscle synergies is not a linear scale from which one can infer efficient movement control [30]. Hence, it is important to consider the loadings within the muscle synergy space to properly understand efficient motor and sensory control; several studies support this notion [12, 18]. These studies have shown that synergies can stay consistent, fractionate, and merge depending on the severity of the neurological condition [32].

In stroke patients, studies show a decrease in the number of synergies over time, but in the loadings, strong correlations between participating muscle groups are observed [19, 50]. This result suggests that stroke patients walk with simpler motor patterns than controls as a compensatory strategy. As stroke patients recover, the complexity of the natural motor behavior is restored, and more components with fewer muscle groups in each pattern are observed. However, in SCI participants, when there is no movement, the activity looks completely random, and therefore, all components with random loadings are needed to describe the majority of the data. As some movement is restored, some coalescence of muscle groups to induce coordinated behavior is observed, which results in a significant reduction in the number of synergies. Although the number of synergies at the end of the study is similar to that of the controls, this result does not indicate that function is completely restored to normal because the muscle loadings are only weakly related to those we observed in normal function. Furthermore, there are constraints regarding the normalization of the sEMG data from the SCI participants in our study. These participants exhibited little to no movement during the trials; therefore, we normalized the $sEMG_{\mathrm{{RMS}}}$ to the largest activity seen within a trial during a follow-up session, which was not much above the baseline noise of the recording devices with minimal movement. We could not normalize the $sEMG_{\mathrm{{RMS}}}$ to the maximum voluntary contraction, as has been performed in stroke studies [50]. Therefore, while opposite changes in the number of synergies between recovery from SCI and recovery from stroke were observed, these are not incommensurate findings.

Moreover, a severe stroke condition results in the merging of synergies in the stroke population, leading to fractionation for improved control [32]. This result can be observed in our study, where synergies associated with BL hip movement and ankle movement are split into individual modules or synergies. This fractionation is shown in Figs. 8 and 9, thus further validating the consistency of our result with the existing literature.

### Functional relevance of muscle synergies

Several studies have concluded that three to five muscle synergies are sufficient to account for the basic patterns of muscle activation in the upper and lower limbs [29, 51, 52]. In our study, the control participants had four muscle synergies on average for each of the BMCA tasks. We observed that the SCI participants required all 10 EMG channels (no synergy) at the beginning of the study, and the number of synergies progressively decreased to 4 or 5 by the final session. While the SCI participants achieved similar numbers of muscle synergies as the control participants, there were significant structural differences in their synergies, which we generally observed during the motor control task. An asymmetry in muscle synergies during BL movements indicates improper inter-leg coordination and has been previously observed in SCI participants [32, 53, 54]. It is clear from our results that persistent tonic stimulation over many months restored muscle synergies across SCI participants, suggesting plasticity in the spinal circuitry.

### Potential neural mechanism of muscle synergies

**Fig. 10:**
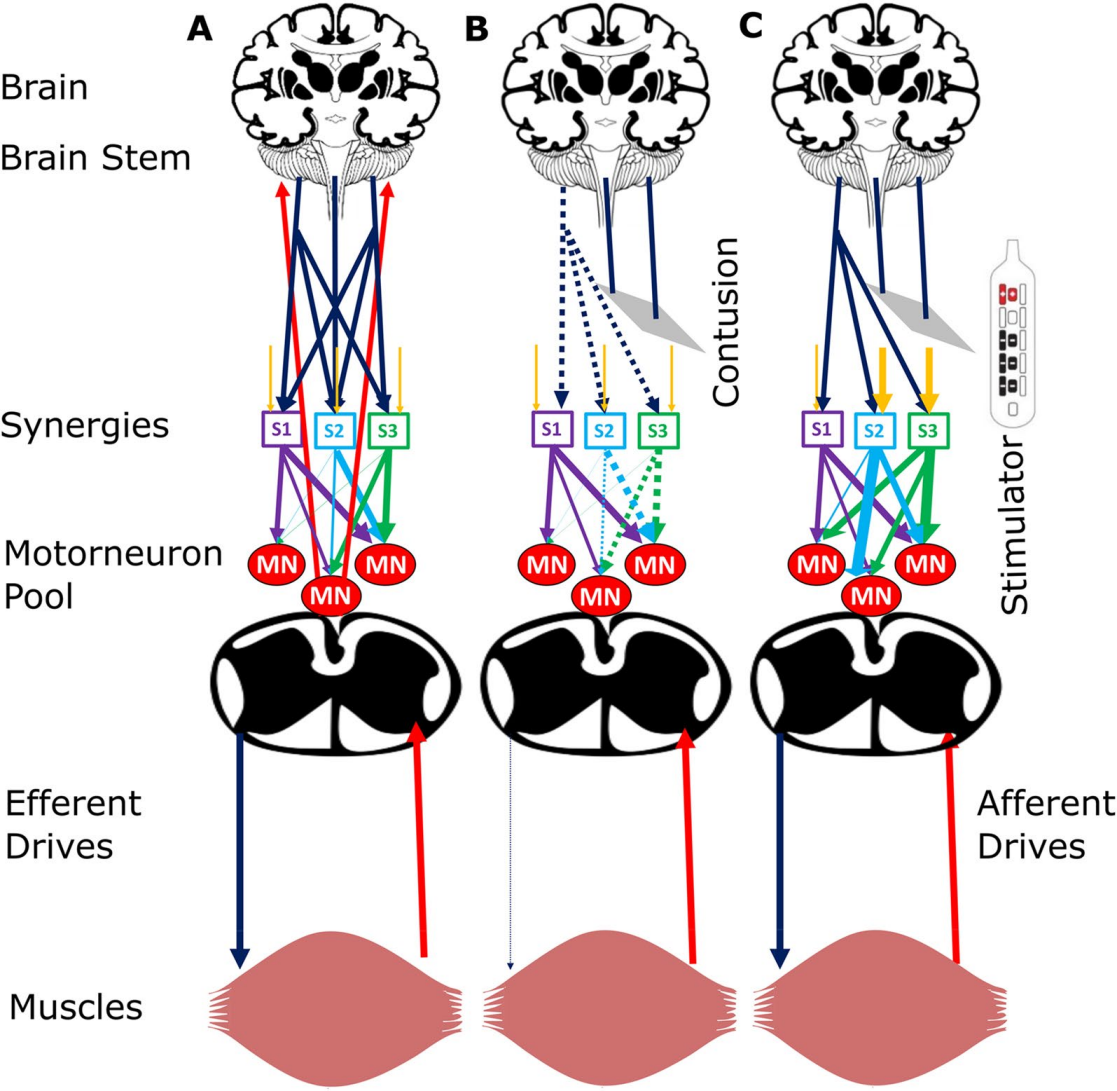
Schematic of hypothetical neural mechanisms. The yellow descending arrows represent local spinal circuitry neural drives, the blue descending arrows represent efferent drives, the multicolored descending arrows between the synergies and motor neuron pool represent the neural network, and ascending red arrows from a muscle represent afferent drives. **A** Neural circuits of intact participants with efferent drives projecting as a network on muscle synergies (S1, S2, S3). The synergies encode the information of the motor neuronal pool (MN) and activate specific groups of muscles to cause movement. The afferent drives, shown as red arrows, bring information from the task to the supraspinal centers, such as the cerebellum and motor cortex, that reduce any error in movement by modulating the efferent drives. **B** Disruption in the spinal circuits after injury obstructs the descending/ascending pathways, causing the inactivation of synergies, which leads to paraplegia. **C** Epidural stimulation modulates the sensory afferent and motor efferent drives within the local spinal circuitry, thereby activating muscle synergies and restoring voluntary movement. Sensory feedback to the supraspinal region is absent in SCI participants

Here, we put forward a hypothesis for how eSCS restores neural synergies, as illustrated in Fig. 10. In control participants, the ascending and descending tracts are intact. During volitional movements, the intact ascending drives act as a task-specific input to the cerebellum, where a predefined model bearing primitive and learned motor behavior exists [49]. The ascending tracts bring proprioceptive information to the cerebellum, where it is compared with existing predefined models for a specific movement. If an error is detected, the movement is corrected by a complex neural network that includes the cerebellum, cerebral cortex, and basal ganglia. This network assists in the modulation of the descending drives. These modulated descending drives then recruit the necessary muscle synergies to correct the movement.

In our study, the SCI participants had motor and sensory complete paralysis, and thus, no sensory information ascended to the supraspinal region. The motor commands from the corticospinal tracts may be severed, but even if a small portion of the spinal cord is left intact, limited cortical signals may project to the spinal cord. The significant reduction in cortical input and drive makes the local spinal circuit unresponsive to these remaining signals, as illustrated in Fig. 10b. eSCS modulates the neural drives within the local spinal circuit so that they become sensitive to these limited cortical or supraspinal signals, which then restores some volitional control to the muscles. In addition, it is also possible that the stimulator modulates the residual corticospinal pathways in the dorsal column rather than actual anterolateral and ventral pathways [55]. These modulated signals further recruit specific muscle synergies to move the limb during a volitional task, as shown in Fig. 10c, thus restoring the muscle synergies and voluntary motor control over time.

While Figs. 8 and 9 show that muscle synergies are restored in eSCS participants, synergies between the sides are not. When the control participants perform BL hip and ankle tasks, their first muscle synergy includes muscles from both the right and left sides, indicating synergy among muscles within a leg as well as synergies between legs. In contrast, the right and left leg muscle synergies are isolated into two groups during hip movement and ankle movement by the SCI participants, indicating a lack of inter-leg coordination. We hypothesize that muscle synergies within a leg occur within the spinal cord, which are restored with eSCS, but synergies between the legs may be more dependent on supraspinal areas, which are not restored, resulting in asymmetric muscle synergy recruitment during BL movement.

### Limitations

One of the limitations of our study is that we did not record the hip extensor muscle groups, which prohibits the analysis of muscle synergies associated with hip extension. The supine posture of the participants made it difficult to record sEMG signals from the hamstrings and gluteus maximus. While sEMG recordings from these muscles would have been useful in the synergy analysis, they were not used because of the high likelihood of the electrodes coming off during the experiment and the noisiness of the signals during BMCA tasks due to movement artifacts.

EMG data were recorded over several sessions to estimate changes in the synergies over time, but because new electrodes were utilized at every follow-up visit, normalization of the amplitudes was needed to compare the muscle activity across sessions. A common approach is to normalize to the maximum voluntary contraction. However, in this study, we were not able to record the maximum voluntary contraction due to the participants’ injuries. Instead, we normalized to the maximum activation of each muscle across all tasks. Previous studies have reported that muscle synergy structures remain consistent across different normalization methods [56]. While normalization to the maximum voluntary contraction would be ideal, we believe that the muscle loadings within synergies estimated from the sEMG data normalized to the maximum activity on each day is an acceptable alternative and is unlikely to affect the findings of this study.

## Conclusion

In this study, we used muscle complexity and synergy analyses to understand the acute and long-term effects of eSCS in participants with chronic motor/sensory complete SCI. Stimulation decreased the muscle activation and localized muscle activity to the rostro-caudal spinal column. It also decreased the muscle complexity, which was estimated using an HDF complexity analysis. Thus, eSCS improves motor function below the level of injury, as observed in the synergy and complexity analyses.

We also observed changes in the coordination of muscle groups over time. The number of muscle synergies decreased over the course of the follow-up sessions, and at the end of 13 visits, the number of synergies required to describe 85% of the muscle activation matched that of the control participants. While a similar number of synergies was observed for the SCI participants with stimulation and the control participants, the muscle loadings within the synergies of the SCI participants did not match those of the control participants. Particularly, when tasks required BL movement, the control participants had BL muscle synergies whereas the SCI participants did not. Overall, our results suggest that epidural stimulation improves movement control by changing the structure and dimensionality of the muscle synergies via acute and chronic neuromodulation.

Finally, this study provided an opportunity to test in humans whether muscle synergies have a neural or task-dependent basis. The restoration of synergies in the same participant performing the same task over time supports the hypothesis that muscle synergies have a neural basis rather than a task basis.

## Supplementary Information

**Additional file 1**. Supplementary Information including Supplementary Tables 1–2 and Supplementary Figures 1–2.

## Data Availability

The data is available from the corresponding author upon reasonable request.
