# Supplementary material for: Epidural stimulation restores muscle synergies by modulating neural drives in participants with sensorimotor complete spinal cord injuries
Source: J Neuroeng Rehabil. 2023 May 3;20:59. doi: 10.1186/s12984-023-01164-1 (PMC10155428; doi:10.1186/s12984-023-01164-1)
Supplement: Supplementary file 1 — Additional file 1. Supplementary Information including Supplementary Tables 1–2 and Supplementary Figures 1–2. [file 12984_2023_1164_MOESM1_ESM.pdf]

Supplementary information

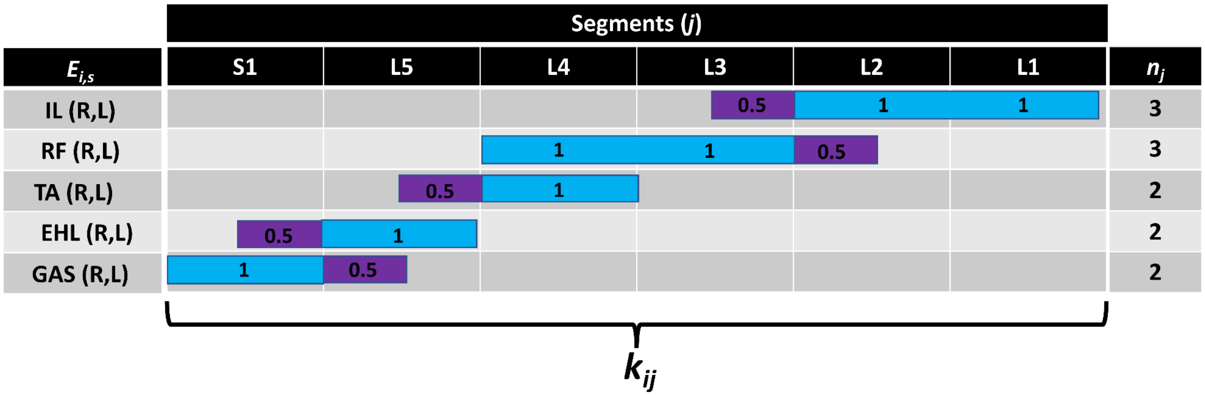

Supplementary Table 1. Sharrard's chart mapping alpha motor neurons at each spinal cord segment to muscles [24]. The table displays weighting (0, 0.5, 1), which are dependent on the number of muscles each spinal segment innervates through alpha motor neurons. This map is used to infer activity in the spinal cord based on the muscle activity measured.

Region of localization in temporal domain as percentage of task completed							
Task	Participant ID	Left		Bilateral (Left, Right)		Right	
		Flexion	Extension	Flexion	Extension	Flexion	Extension
Hip	SCI 001	16-50%	NA	17-40%, 6-40%	NA,NA	11-40%	NA
	SCI 002	25-50%	NA	12-25%, 6-37%	NA, 55-75%	NA	60-80%
	SCI 003	8-16%	NA	4-18%, 6-15%	NA,NA	8-16%	NA
	SCI 004	6-18%	NA	18-23%, 11-23%	NA,NA	NA	75-77%
	SCI 005	6-11%	NA	3-7%, 5-38%	NA,NA	5-20%	NA
	SCI 006	10-31%	NA	14-43%, 14-42%	NA,NA	15-40%	NA
Ankle	SCI 001	NA	70-90%	22-50%, 21-50%	NA,NA	NA	50-70%
	SCI 002	18-20%	NA	18-40%, 70-80%	70-80%, NA	NA	75-87%
	SCI 003	10-20%	NA	11-50%, 12-48%	NA,NA	5-14%	NA
	SCI 004	15-20%	75-80%	81-95%, 85-95%	NA,NA	26-30%	NA
	SCI 005	22-32%	NA	31-37%, 14-33%	NA,NA	47-63%	NA
	SCI 006	NA	NA	29-44%, 29-50%	NA,NA	16-45%	NA

Supplementary Table 2. In control patients, there is a clear distinction between flexion and extension of the hip or ankle when patients are instructed to move. This table shows the average percentage of the trials in which SCI participants generated flexion and extension movement, as determined from the spinal maps. The table shows activity for each patient across left, right and bilateral for hip and ankle movements. Most SCI participants only generate significant muscle activity during a single phase of the movement.

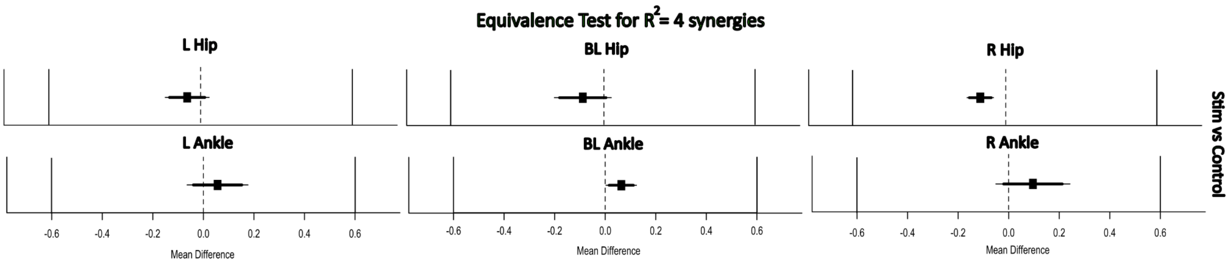

Supplementary Figure 1. Equivalence test of synergies between control and SCI participants. To determine if SCI participants had a statistically similar number of components as controls, we used the independent groups equivalence test between SCI patients without stimulation on their last follow-up session and controls. The equivalence test was applied to measure the cumulative R^2 value of the first four synergies/components from SCI and controls. We used the fourth component because it is generally about the number of synergies required to determine 85% of the total variance. The equivalency test shows that the total cumulative variance explained by the first four components is statistically not different between SCI and control participants for the majority of the BMCA tasks. There are small but significant differences in the R Hip and BL Ankle synergies. However, the test shows that R^2 value for the four synergies between SCI without stimulation and controls is equivalent for the BMCA tasks.

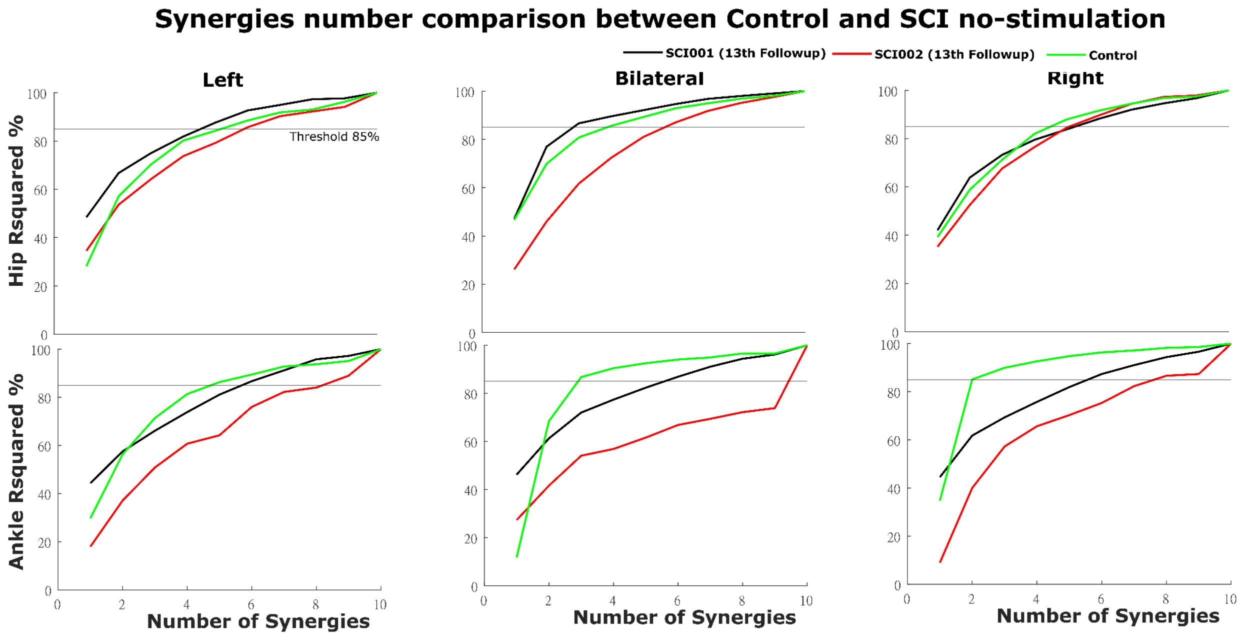

Supplementary Figure 2. R^2 curve for two SCI participants whose volitional motor functions were restored is compared against control. The SCI001 and SCI002 on the 13th follow-up session were able to display movement control in the absence of stimulation. The R^2 values were much closer to that of control participants compared to their first follow-up session without stimulation. Moreover, the curves for SCI001, SCI002, and control are quite indistinguishable during unilateral hip movement. Thus, indicating that eSCS therapy without optimized stimulation settings improves outcomes, as shown in Figure 5 (main article).
